# Independent Demographic Responses to Climate Change among Temperate and Tropical Milksnakes (Colubridae: Genus *Lampropeltis*)

**DOI:** 10.1371/journal.pone.0128543

**Published:** 2015-06-17

**Authors:** Sara Ruane, Omar Torres-Carvajal, Frank T. Burbrink

**Affiliations:** 1 Department of Biology, College of Staten Island, 2800 Victory Blvd., Staten Island, NY, 10314, United States of America; 2 The Graduate Center, City University of New York, 365 5^th^ Avenue, New York, NY, 10016, United States of America; 3 Museo de Zoología, Escuela de Biología, Pontificia Universidad Católica del Ecuador, Avenida 12 de Octubre y Roca, Apartado 17-01-2184, Quito, Ecuador; State Natural History Museum, GERMANY

## Abstract

The effects of Late Quaternary climate change have been examined for many temperate New World taxa, but the impact of Pleistocene glacial cycles on Neotropical taxa is less well understood, specifically with respect to changes in population demography. Here, we examine historical demographic trends for six species of milksnake with representatives in both the temperate and tropical Americas to determine if species share responses to climate change as a taxon or by area (i.e., temperate versus tropical environments). Using a multilocus dataset, we test for the demographic signature of population expansion and decline using non-genealogical summary statistics, as well as coalescent-based methods. In addition, we determine whether range sizes are correlated with effective population sizes for milksnakes. Results indicate that there are no identifiable trends with respect to demographic response based on location, and that species responded to changing climates independently, with tropical taxa showing greater instability. There is also no correlation between range size and effective population size, with the largest population size belonging to the species with the smallest geographic distribution. Our study highlights the importance of not generalizing the demographic histories of taxa by region and further illustrates that the New World tropics may not have been a stable refuge during the Pleistocene.

## Introduction

Populations of all extant species that originated prior to the Holocene have experienced cyclical climatic change. For those found in temperate regions of North America and Europe, populations likely responded directly to climate changes induced by the glacial and interglacial cycles of the Quaternary [1]. Populations of different species occurring in areas of changing environments may each respond uniquely and experience either size decreases or increases, or simply remain stable. Some taxa, such as mammoths and other large mammals, declined in abundance, and ultimately became extinct [2]. Others persisted by adapting to the new environments [3] or, for instance during the Last Glacial Maximum (LGM), by migrating to more suitable areas [3,4]. Although some species persisted through periods of climatic change, population sizes may have been drastically reduced (e.g., [5]). In contrast, when conditions become favorable, population sizes for some species increased rapidly (e.g., [6–9]; reviewed in Hewitt [4,10–12]).

The signature of these demographic changes can be detected from genetic data analyzed with coalescent-based models (reviewed in Ho and Shapiro [13]). Trends with respect to changes in effective population size (*N*_e_) through time can be associated with climatic or geological phenomena which may have altered population sizes or structure [14–21]. Demographic studies have until recently relied heavily on a single locus. Unfortunately, single loci may not represent the demographic history of the species or population under examination, especially if there has been selection on that particular gene region [22]. For example, balancing selection results in an excess of intermediate-frequency alleles for a locus, giving a genetic signature that is indistinguishable from population contraction [23,24]. Conversely, it may not be possible to separate the effects of purifying selection from population expansion, as both yield an excess of rare alleles [23,24]. These confounding effects of selection are therefore avoided by using multiple, unlinked markers (e.g., [25]).

For Nearctic species, Pleistocene glacial cycles may have directly altered distributions and populations sizes, where cycles of decline and growth are expected to occur with the contraction and expansion of favorable habitat, respectively [20,26–29]. The most recent glacial event in North America, the Wisconsin, began ~120–80 kya and reached its maximum at ~20 kya [30], with retreat at ~10 kya marking the end of the Pleistocene and beginning of the Holocene. During the LGM, much of eastern North America (NA) was covered by ice sheets as far south as the 38° latitude in some areas (Illinois, Indiana, and Ohio [31]). Cooler temperatures and boreal forests dominated much of the eastern U.S., pushing hardwood forests much farther south [32]. Open pine-forest habitat was predominant across western NA and the deserts of the southwest were cooler and wetter compared to current climates [33]. Several studies have found that after the LGM, temperate North American species expanded as more suitable habitat became available (e.g., pitvipers [9], woodpeckers [34], and rodents [35]). These climate changes may have also contributed to the diversification of species by isolating once contiguous populations into separate southern refugia [4,11,12,36,37].

The same glacial cycles may also be important for speciation and population size changes in the Neotropics [38–40], yet studies examining historical demography in the species-rich tropical biota of the New World (NW) are lacking (reviewed in [37]). In contrast to the impacts that glacial cycles had on Nearctic species, the effects on Neotropical taxa may not have been as severe [7] given the lack of extensive ice sheet formation in the tropics. Earlier studies suggested that the Neotropics were a region of climatic stability during the Pleistocene [41–43], but some studies now demonstrate that significantly lower temperatures by ~4–9°C were common across the NW tropics during the LGM [44–52]. Additionally, glaciers existed in high altitude regions of Mexico, Guatemala, Costa Rica, and in the Andes Mountains of South America during this time [53–56], with evidence of Pleistocene cooling in both Central and South America and increased aridity persisting until the start of the Holocene [11,12,51,57]. Climate alterations subsequently affected plant distributions [51], causing high altitude vegetation to shift to lower elevations and reducing tropical rain forest while increasing grasslands in Central and South America (reviewed in [11,12,51]). These habitat changes may have altered population dynamics of species inhabiting tropical rainforests, but the demographic impacts of these changes have not been extensively explored. Time-calibrated phylogenetic studies that include tropical species indicate that diversification took place during the Pleistocene for some taxa [58–63], but geological events throughout the Miocene and Pliocene also contributed to speciation in both Meso and South America [61,63–67]. Additionally, Quaternary climatic events may have affected population demographics of taxa, as they did with temperate species.

Although there are numerous examples of vertebrate clades that have representatives in both the NW temperate and tropical regions, no studies have directly compared the historical demography between such taxa. The snake genus *Lampropeltis*, commonly known as the kingsnakes and milksnakes, is composed of several closely related species in both temperate North America and tropical Central and South America. A recent study demonstrated that diversity within *Lampropeltis*, specifically within milksnakes, was underestimated, indicating that the former single species of milksnake, *L*. *triangulum*, is composed of seven taxa distributed in North, Central, and South America [62]. Therefore, we propose to examine the effects of Quaternary climate change on six closely related taxa that are found in both Nearctic (N = 3 species) and Neotropical (N = 3 species) regions. The three temperate species (*L*. *triangulum*, *L*. *gentilis*, and *L*. *elapsoides*) are found primarily within the United States, with one extending into southeastern Canada, and are found across a variety of habitats, which include deciduous forest, pine forest, plains, and desert conditions [68,69]. The three tropical species (*L*. *abnorma*, *L*. *micropholis*, and *L*. *polyzona*) are distributed throughout Central America and northern South America, with their very southern limit in Ecuador. These three species are found in thornscrub and in wet and dry tropical forest, as well as in both lowland and highland habitats [69–71]. Using a multilocus dataset, we test whether temperate North American species of milksnake had similar demographic responses compared to tropical Central/South American species. Specifically, we examine changes in population size through time for each taxon to determine if temperate and tropical species show similar demographic trends throughout the Pleistocene. We hypothesize that temperate species will show evidence of declines leading up to/and or during the LGM due to habitat contraction into southern refugia. This model suggests that temperate species, two of which have a current range that extends beyond the former southern extent of the glacier (*L*. *triangulum*, *L*. *gentilis*), would have then experienced range expansion after the retreat of the Laurentide Ice Sheet and increase in available habitat, as has been reported for other North American taxa [7].. Conversely, the tropical species may have maintained stable population sizes throughout the recent Pleistocene as has been shown for Amazonian mammals [7]. However we also expect to see increases in tropical milksnake population sizes around the beginning of the Holocene, which would be associated with the favorable environmental conditions present today.

By examining milksnakes across their extensive range in a historical demographic context, we address how population structure has been altered by Pleistocene climate change in closely related Nearctic and Neotropical taxa. We ask if there are general unified responses to climate change for closely related species. If so, these responses suggest that closely related taxa experience similar population increases or decreases regardless of location given changing climates. In contrast, it is possible that specific historical demographic trends are reflective of climate change associated with a specific region. In this case, we would expect that the population histories of species would be similar based on geographic ranges, with temperate species sharing similar demographic responses and tropical species sharing similar demographic responses).

## Methods

### Sampling and Genes

**Fig 1:**
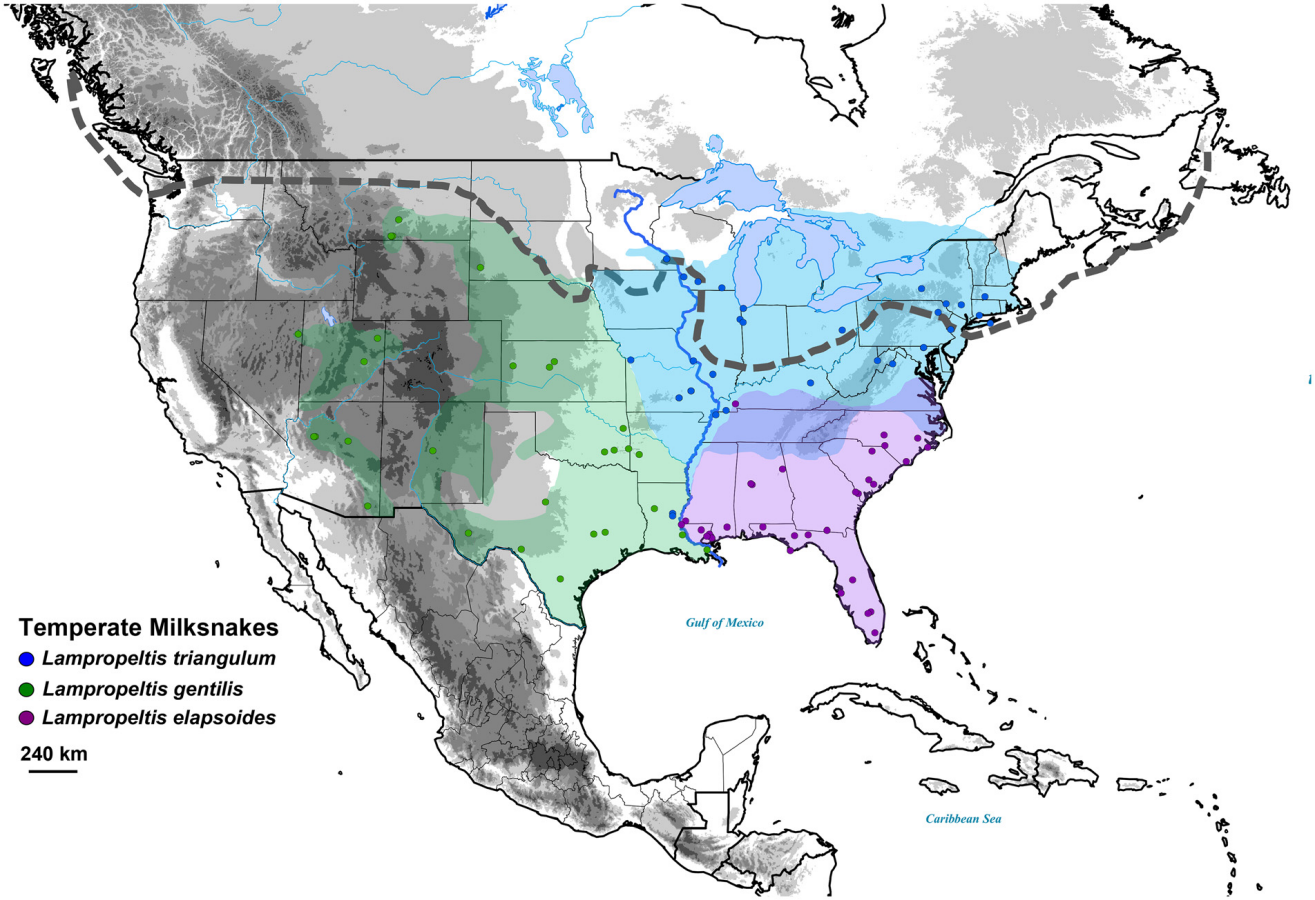
Map showing sampling, and estimated full ranges of three temperate milksnakes, *Lampropeltis triangulum* (blue), *L*. *gentilis* (green), and *L*. *elapsoides* (purple). The extent of the Laurentide Ice Sheet during the last glacial maximum is shown by the grey dotted line (adapted from Pielou [85]). Ranges of all species are based on Ruane *et al*. [62]

**Fig 2:**
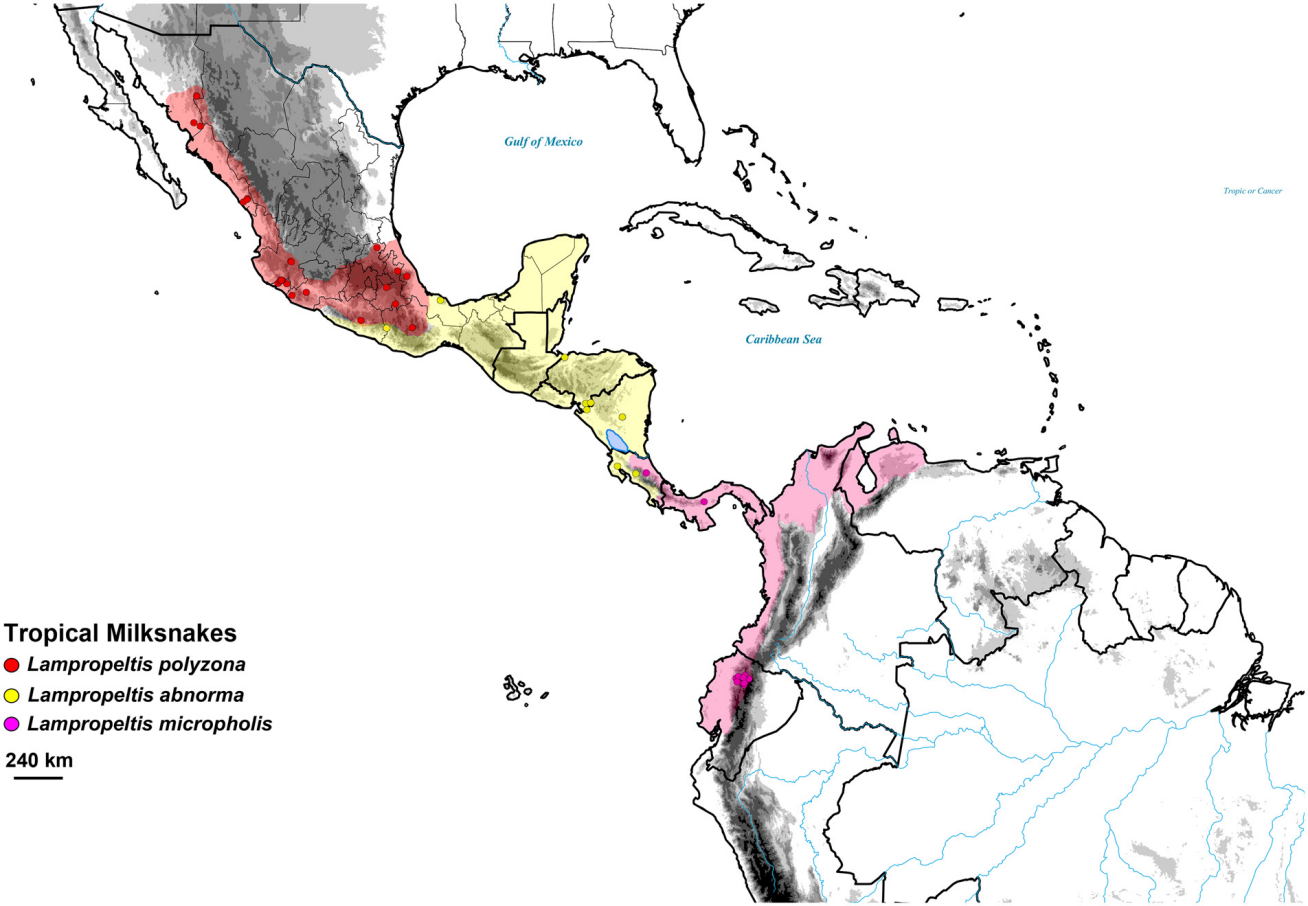
Map showing sampling, and estimated full ranges of three tropical milksnakes, *Lampropeltis polyzona* (red), *L*. *abnorma* (yellow), and *L*. *micropholis* (pink). Ranges of all species are based on Ruane *et al*. [62].

We selected six milksnake species for our analyses of demographic change; *L*. *triangulum*, *L*. *gentilis*, *L*. *elapsoides*, *L*. *polyzona*, *L*. *abnorma*, and *L*. *micropholis*, following the revised taxonomy of Ruane et al. [62] and using GenBank sequences from accession series KF214996–KF216452. These six taxa, with an abundance of populations previously sampled, have extensive distributions throughout the Nearctic and portions of Neotropics (Figs 1 and 2). The three temperate species included *L*. *triangulum* (*n* = 34), *L*. *gentilis* (*n* = 30), and *L*. *elapsoides* (*n* = 32), occurring in the USA and Canada. The tropical species included *L*. *polyzona* (*n* = 31), *L*. *abnorma* (*n* = 11), and *L*. *micropholis* (*n* = 16), rangeing from Western/Central Mexico to Ecuador (see S1 Table in supporting information for details of samples). All six species originated between 1.1 and 3.6 Ma [62] and so would have experienced Late Pleistocene climate change. For *L*. *triangulum*, *L*. *elapsoides*, and *L*. *gentilis*, three genes were used, the mitochondrial gene cytochrome b (Cytb) and two anonymous nuclear loci, 2CL8 and CL4. For *L*. *polyzona*, *L*. *abnorma*, and *L*. *micropholis*, the same three genes were used; additional nuclear loci were available for the tropical taxa as well and so we also included the protein coding genes NT3 and PRLR, the introns SPTBN intron 1, Vimenton intron 5, NAV intron 5, and Z-chromosome GAD intron 15, and the anonymous loci 2CL3, 2CL4, and LATCL (S2 Table). Nuclear loci heterozygosities were previously resolved with PHASE v2.1.1 [72] in Ruane et al. [62], and so we used the most probable pair of resulting alleles for analyses.

### Historical Demographic Analyses

To determine if temperate and tropical milksnakes show similar or distinctly different trends with respect to demographic histories, we examined population size changes through time using Extended Bayesian Skyline Plots (EBSP; [73]) implemented in BEAST v.1.7.2 [74]. Using a multi-gene coalescent approach, this method estimates population size through time and does not require a pre-specified demographic model. We ran the EBSPs using all available loci, as well as using only the nuclear loci to determine if mtDNA had a large influence on the results. For the dataset including all loci, we used the mean substitution rate of Cytb estimated for *Lampropeltis* of 1x10^-8^ to scale the time axis, following Pyron and Burbrink [75]. For the nuclear only EBSPs, we used the mean rate estimated for the locus 2CL8, based on the full loci analyses (3.7x10^-10^). The EBSP for each species was run between 2x10 ^8^ to 5x10^8^ generations to achieve high effective sample size (ESS) values. Stationarity was assessed using Tracer v.1.5 [76]. We examined the demographic populationSizeChange parameter in Tracer, which indicates the most probable number of population size changes for each species. To determine if higher *N*_e_ (based on using the full loci datasets) was correlated with range size, we calculated the approximate range size in square kilometers in Google Earth Pro using the polygon function (www.googleearth.com) for each species, with the ranges based on Ruane et al. [62] (complete species range) as well as using the minimum polygon spanning only the samples used here (sample species range). We then used a Spearman rank correlation test to determine the relationship between range size and mean *N*_e_. We also used this test to determine if the net population size change (the difference between the *N*_e_ at start of the EBSP and the *N*_e_ at time zero) and range were correlated. Spearman rank correlations were performed in the statistical program R [77].

We also tested for population size changes using non-genealogical coalescent methods (i.e., these methods do not reconstruct the genealogy of sequences) to compare estimates for each locus independently. These tests help address the confounding effects of changes on *N*_e_ versus selection upon a particular locus. We used the program DNAsp 5.0 [78] to examine population size changes using several summary statistics. The *R*_2_ statistic [79] was used to test for population expansion and is based on the difference in the number of singleton mutations and the average number of pairwise differences between samples. Coalescent simulations implemented in DNAsp were then used to assess significance and confidence intervals. We also generated mismatch distributions; resulting distributions indicate changes in population size or selection [80]. Harpending’s raggedness index (*r*_*g*_, [81]) was then used to assess the statistical significance of these distributions. Additionally, Tajima’s D [82] and Fu and Li’s D* and F* statistics [83] were used to test for population growth or decline versus constant population size. For these three statistics, an overabundance of rare polymorphisms (resulting in negative values) indicates there has been population expansion or positive selection, while positive values, resulting from an overabundance of intermediate-frequency polymorphism, indicate population decline or balancing selection.

## Results

### Sampling and Genes

For the EBSP analyses including all loci, all sequences were included, with 3–11 polymorphic loci for each species and 11–34 individuals per locus (S2 Table). For the nuclear only EBSP analyses, the same loci, excluding Cytb were included with 2–10 polymorphic loci for each species and 11–34 individuals per locus (S2 Table). For DNAsp analyses, sequences missing a significant amount of data for a gene were removed from analyses, as DNAsp excludes all sites with missing data (S2 Table).

### Demographic Analyses

**Fig 3:**
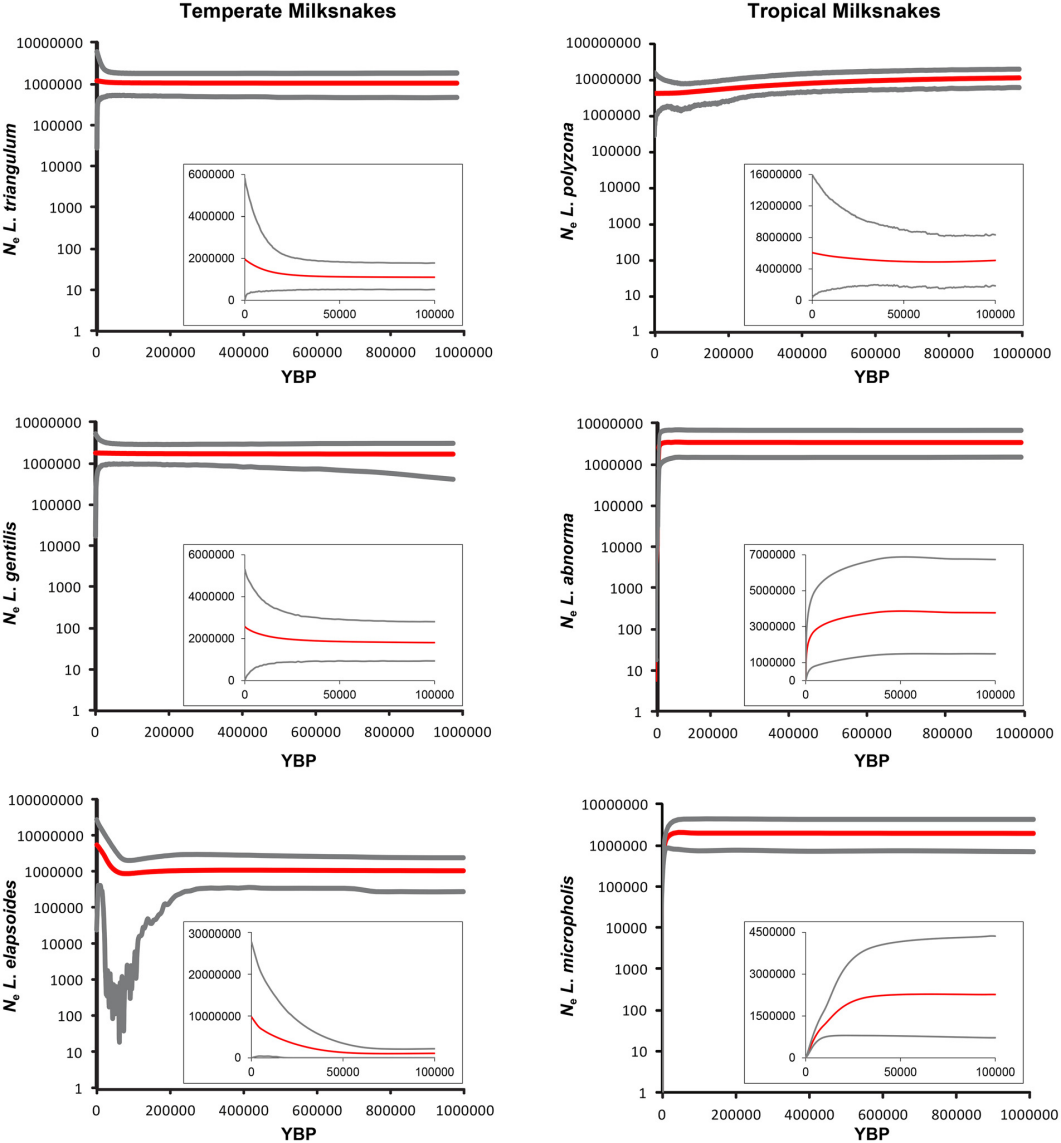
Extended Bayesian Skyline Plots for each species of milksnake for the last one million years before present (YBP), with a zoomed inset of the last 100,000 YBP, showing the median (black) and 95% highest posterior density changes in effective population size (*N*_e_) over time on a log-transformed scale.

We recovered high ESS values (>200) for all parameters in BEAST and for every taxon, with the exception of *L*. *micropholis*. Despite increasing the chain length for *L*. *micropholis*, the ESS values for some parameters (e.g., the prior) did not greatly improve and so multiple runs were used to ensure consistency among results. The EBSPs using all loci generally showed stable populations through time for *L*. *triangulum* and *L*. *gentilis*, but for *L*. *elapsoides*, there was an increase in population size at ~50 kya (Fig 3). In contrast, the two most southerly distributed species, *L*. *abnorma* and *L*. *micropholis*, showed a slight population increase at ~70 kya and then a decline starting ~45 kya continuing to the present (Fig 3). The third tropical species, *L*. *polyzona*, showed a long period of population decline starting at least 1 Ma, followed by a slight increase and population stability beginning ~80 kya (Fig 3). Mean *N*_e_ ranged from ~1,100,000 (*L*. *micropholis*) to ~10,000,000 (*L*. *polyzona*; Table 1). The temperate species had fewer detectable population size changes (mean/median number of changes = 0.5−1.6/0.0−2.0; 95% HPD = 0.0−3.0 changes across species) compared to the tropical species (mean/median number of changes = 2.0−2.6/2.0; 95% HPD = 2.0−4.0 changes across species; Table 1). Overall, the temperate species also had larger population sizes and larger ranges than did the tropical species, with the exception of *L*. *polyzona*, which had the largest population size and the smallest complete species range (Table 1). However, the Spearman rank correlations for both complete range size versus mean *N*_e_ (*r*_s_ = -0.029) and complete range size vs. net *N*_e_ (*r*_s_ = 0.086) were not significant (d. f. = 4; *P* > 0.05), nor were the sample range sizes versus mean *N*_e_ (*r*_s_ = 0.37) and sample range size vs. net *N*_e_ (*r*_s_ = 0.20); (d. f. = 4; *P* > 0.05). When the EBSP analyses were run using only the nuclear loci, results were similar to the full loci EBSPs for some species for the populationsizechange parameter (*L*. *polyzona*, *L*. *micropholis*), but only *L*. *micropholis* resulted in a non-zero HPD (mean/median = 2.0/2.0; 95% HPD = 2.0−2.0 changes). Being that the EBSP estimates for the nuclear only datasets had larger posterior distributions for both population sizes and the population size change parameter and that these results were likely less accurate than those including all available data (S3 Table), we do not discuss them further here and instead rely upon the EBSPs that include all the loci. The results from tests performed in DNAsp (the *R*_2_ statistic, mismatch distributions, Tajima’s D, and Fu and Li’s D* and F* statistics) did not reject the scenarios of neutrality/population stability for *L*. *triangulum*, which indicate no detectable selective or demographic forces operating on this species. For the other five species, no clear trend was detected across any of the loci for any species using these summary statistics (S3 Table). Although several loci resulted in positively and negatively significant test results, these results were not consistent across loci for a given species and in several cases, different loci for the same species indicated different patterns (S4 Table).

**Table 1:** Approximate complete range size, sample range size, mean and median effective population size and number of size changes for six species of *Lampropeltis*.

Species	Complete Range Size	Sample Range Size	Population Size (Millions) Mean, Median (95% HPD)	Number of Population Size Changes Mean, Median (95% HPD)
*L*. *triangulum*	1,800,400 km^2^	600,100 km^2^	5.122, 1.558 (6.385 x 10^4^–1.564 x 10^7^)	0.5, 0.0 (0–2)
*L*. *gentilis*	1,700,000 km^2^	900,300 km^2^	9.547, 2.389 (1.104 x 10^5^–2.559 x 10^7^)	0.6, 0.0 (0–2)
*L*. *elapsoides*	900,000 km^2^	460,100 km^2^	7.950, 3.463 (1.168 x 10^5^–2.260 x 10^7^)	1.6, 2.0 (0–3)
*L*. *polyzona*	400,400 km^2^	240,000 km^2^	9.777, 7.057 (1.509 x 10^6^–2.428 x 10^7^)	2.6, 2.0 (2–4)*
*L*. *abnorma*	750,400 km^2^	290,000 km^2^	1.929, 1.379 (2.060 x 10^5^–5.034 x 10^6^)	2.0, 2.0 (2–2) *
*L*. *micropholis*	560,200 km^2^	61,000 km^2^	1.108, 7.638 (1.044 x 10^5^–3.128 x 10^6^)	2.0, 2.0 (2–2) *

The 95% highest posterior density (HPD) is shown for each in parentheses; for number of size changes, species that had non-zero HPDs are indicated (*)

## Discussion

Whether glacial cycles have had similar effects on population sizes in both temperate and tropical regions is of interest for two reasons. First, it provides insight into the factors that have shaped the current demographic patterns and distributions of organisms in disparate environments and helps determine whether closely related taxa respond similarly regardless of location. Second, from a conservation perspective, understanding how past climate change affected populations or taxa may be applicable to determining what threats species face when confronted with anthropogenic climatic alteration. Here, we find that for milksnakes in the genus *Lampropeltis*, there is no shared demographic pattern with respect to temperate versus tropical species, although tropical species show less population stability when compared to the temperate species. Based on the EBSPs, populations of temperate species have either remained generally stable or increased in size during the recent Pleistocene/Holocene (Fig 3). This stability is surprising, considering that the LGM would have had a direct impact on snakes living in temperate North America, with the Laurentide Ice Sheet covering a proportion of the ranges of both *L*. *triangulum* and *L*. *gentilis* (Fig 1; [31,84,85]). The three tropical taxa showed population expansion as well as declines (Fig 3), which in part supportsthe hypothesis that tropical environments did were not stable throughout the last glacial period.

Previous work has demonstrated that tropical species tend to have smaller geographic ranges compared to temperate species and that with smaller ranges come smaller population sizes (reviewed in [86–88]). Some studies have also shown that for snake lineages, those with larger ranges generally have a higher *N*_e_ (e.g., [8]), although this trend is not consistent among taxa (e.g., [89]). Despite a general lack of correlation between range size and population size in milksnakes, we do find the temperate species did have greater ranges and larger mean effective population sizes. The exception to this pattern was the tropical *L*. *polyzona*, which had the largest mean population size despite having the smallest complete species range (Table 1; Figs 1 and 2). There is the possibility that individuals with the greatest amounts of genetic variation were inadvertently sampled, which would result in signal that indicates large population sizes [90]. This potential bias could be corrected by increasing sample sizes, which would better reflect the actual allelic variation within *L*. *polyzona* and result in a more accurate estimate of population size. However, if *L*. *polyzona* represents a single, panmictic species, as is indicated by the results of a prior study [62], the large *N*_e_ is likely an accurate estimate, as the sample size for this species (*n* = 31) should be sufficient for detecting population size changes [73]. Although most studies on historical demography only consider the effects of climate change on *N*_e_, it is important to note that there are other plausible biotic reasons for changes in historical population size that should not be dismissed, such as competition between species or predation (reviewed in [91]). Unfortunately, there is no clear way to separate the effects of biotic interactions from climate change on *N*_e_ through time in these snakes and so we limit our discussion to climatic or geological events within the time frame considered here.

### EBSP Results and Summary Statistics

We find a lack of concordance between the EBSPs and the non-genealogical summary statistic results. The summary statistics for many loci did not indicate the same demographic signal as the EBSP trends did, resulting in conflicting signals or a lack of significance (S4 Table). The exception to this is *L*. *triangulum*, which showed no significant *N*_e_ changes for any non-genealogical test across loci and demonstrated stable population sizes via EBSP (Fig 3, Table 1). For the remaining species, the degree to which the summary statistics disagreed with the EBSP trends was variable. For example, one locus for *L*. *abnorma* was significantly negative and indicated expansion (PRLR, *R*_2_; S4 Table), another was significantly positive for population decline (SPTBN1, Fu and Li’s D*; S4 Table) and the remaining nine loci were neutral for all tests (S4 Table). In contrast, the EBSP showed a population decline beginning ~45 ka. These results may indicate there is either balancing or purifying selection acting on some of the loci as the signal cannot be detected across all or most of the loci and would explain the conflicting results. However, for the majority of loci that are not significantly different from neutral, it is possible the summary statistics cannot adequately detect subtle population size changes. Summary statistics may not provide as realistic an estimate of historical population size changes compared to methods that are based on a coalescent genealogy [90]. Previous studies have recognized that *N*_e_ estimates that rely on the calculation of pairwise differences and segregating sites are inefficient at estimating population demographics when compared to methods that account for population trees [92]. EBSPs in contrast generate both demographic signal and coalescent history across all loci simultaneously and provide an estimate of the phylogenetic error associated with the data [13]. Ideally, all loci should retain demographic signature, but events such as extreme bottlenecks can erase signal at a given locus; when using multiple loci given varying substitution rates, there is a chance that some loci are preserving signal lost by other genes [13]. With respect to loci and lost signal, we note our demographic reconstructions are likely influenced most by the highly variable mtDNA locus included, as the results for each species when using only the nuclear loci have larger 95% HPDs for both *N*_e_ and the population size change parameter, and generally predict much smaller population sizes for all species (Appendix 3). However, discounting the additional information provided through the inclusion of multiple independent loci in these analyses would be imprudent as any given locus, even those with slower substitution rates, may provide demographic signature that may not be recovered by other loci. Furthermore, in cases where population growth rates are slow and/or size changes are small, genealogical methods should outperform pairwise methods [93,94]; this is likely important here since the EBSPs for several milksnakes reveal only moderate changes in population size (Table 1; Fig 3). Although simulation-based studies have found that changes in population sizes using Bayesian skyline plots, particularly with respect to declines, may be occasionally incorrect, we note that the sampling strategy used here is most similar to that which was shown to perform best with respect to detecting actual demographic changes (“pooled sampling” [95]). Considering the overall robustness associated with genealogical methods using coalescent models and specifically those using multiple loci [90,94], we base our discussion on the EBSP results rather than the summary statistics.

### Temperate Milksnakes

For two of the temperate species we found no discernible changes in the population size change parameter (both species mean/median size changes < 1.0/0.0; 0.0−2.0 = 95% HPD; Table 1) although the EBSP does show some increase in population size for *L*. *triangulum* ~18 kya (Fig 3). *Lampropeltis triangulum* inhabits deciduous forests of eastern North America, as far north as southern Ontario, and the transition from eastern deciduous hardwood forest to the western prairies coincides with the limit of its distribution (Fig 1; [68]). The equally wide-ranging *L*. *gentilis* is frequently found in grassland or prairie habitat [68,69] west of the Mississippi River, from Arizona to Montana (Fig 1). These two species may have moved into suitable habitat that was pushed further south by the Laurentide Ice Sheet and population sizes therefore remained overall constant, with some possibility of expansion for *L*. *triangulum* after the LGM. In addition, while *L*. *gentilis* is often associated with prairies and grasslands, it can be found in desert lowlands, pine and hardwood forests, and stream valleys [68,69]; the ability of *L*. *gentilis* to inhabit multiple habitat types may indicate it was able to readily adapt to climatic changes of the Pleistocene and thus population sizes remained stable [96]. Other studies of temperate North American snakes have found similar patterns of stability throughout the Pleistocene (e.g., [8,9,97]).

Unlike the two most northern temperate milksnakes, the southeastern U.S. milksnake *L*. *elapsoides* underwent at least one population expansion (mean/median size changes = 1.6/2.0; 0.0–3.0 = 95% HPD) that can be visualized on the resulting EBSP ~50 kya (Fig 3). *Lampropeltis elapsoides* is found in the pine forests of the southeastern coastal plain, throughout the Florida peninsula, and west to eastern Louisiana (Fig 1). The onset of the population increase occurred during the middle of the Wisconsin glacial interval (Fig 3; [98]), and continued to the present. A “thermal enclave” from warm ocean winds throughout the southeastern U.S. during the Pleistocene may have facilitated this population growth [99]. It is also possible that *L*. *elapsoides* shifted its distribution and populations subsequently expanded, as species inhabiting the eastern U.S. may have moved south into both peninsular Florida and Mexico during periods of glacial cooling and returned north once climates became more favorable, as has been proposed for multiple species [100].

### Tropical Milksnakes

Our results indicate that one of the tropical milksnake species increased its population size during the last 100 kya, while the two remaining taxa experienced population declines, suggesting that environmental conditions may not have been stable throughout the Neotropics. At ~80 ka, the EBSP for the Mexican milksnake *L*. *polyzona* shows increasing population sizes(Fig 3). While the mean number of size changes for this species indicates that it has likely undergone several population size changes (mean/median number of size changes = 2.6/2.0; 2.0−4.0 = 95% HPD), we focus on this most recent size change here pertinent to understanding how recent climatic change impacts population demography. This species has a range that abuts several major geological features of Mexico, including the Sierra Madre Occidental, the Sierra Madre del Sur, the eastern versant of the Sierra Madre Oriental, and throughout the Trans-Mexican Volcanic Belt region (Fig 2), and the presence of glaciers in these montane regions during the Wisconsin glacial may have influenced population demography [54]. However, the *L*. *polyzona* populations showed no decline during the late Quaternary as might be expected. It has been proposed that despite glacial presence in northern and central Mexico, climate did not change significantly [101] and has been consistent during the last 30 kya [102], although arguments against stability have been made [54]. The range of *L*. *polyzona* also appears tightly correlated with that of Mexican tropical dry forest, which appeared prior to the origin of this species of milksnake [62,103]. If this habitat remained stable during the Pleistocene, the *L*. *polyzona* population may have persisted and expanded. Interestingly, although there are few studies that examine historical demography for organisms specifically in this region, one of the few species studied that has a similar range in Mexico, the jaguar, shows genetic signal indicating recent population expansion [104], as does a recent study on *Boa constrictor* in that region [105]. Additional demographic studies that focus on this region would help determine whether Mexican dry tropical forest was a stable habitat during the late Quaternary.

In contrast to *L*. *polyzona*, both *L*. *abnorma* and *L*. *micropholis* have experienced population declines beginning ~45 kya (Fig 3), although similar to *L*. *polyzona*, these species likely underwent more than a single population size change (both species mean/median number of size changes = 2.0/2.0; 2.0−2.0 = 95% HPD). The areas occupied by both species are thought to have experienced significant cooling and increased aridity during the Pleistocene. *Lampropeltis abnorma* inhabits much of Mesoamerica, from south of the Sierra Madre del Sur in Mexico, and throughout Central America as far south as Costa Rica, while *L*. *micropholis* occurs in eastern Costa Rica, Panama, and Colombia, Ecuador, and Venezuela north/west of the Andes (Fig 2). Glaciation of the Talamanca cordillera of Costa Rica and the highlands of Guatemala and Mexico during the Wisconsin contributed to the cooling of Central America [53,56], which may have started as early as 45 kya [106,107] potentially resulting in the decline of *L*. *abnorma* populations. While it is suggested that deglaciation occurred ~11 ka, aridity throughout Central America was at its maximum at this point in time [107]. Snakes are sensitive not only to changes in temperature but also precipitation, with drier conditions potentially limiting the distribution of a species [108]. Like *L*. *abnorma*, *L*. *micropholis* populations may have been influenced by the glaciers in Costa Rica as well as the changing climate of lower Central America, which was cooler and drier throughout much of the last 100 kya [109]. The extent of its range also means *L*. *micropholis* may have been impacted by Andean glaciations during the Pleistocene. As glaciers expanded across the Andes [55] vegetation moved with respect to elevation [106,110], and similar to Central America, South American climates became drier and tropical forests decreased [45]. This may have restricted the size of the habitat available for *L*. *micropholis* and caused the population to decline during the last 45 kya. Although the NW tropics were initially thought to be a stable region during recent glacials [7,41–43], the response patterns for the three tropical milksnakes examined here are more similar to studies that show disparate demographic histories for closely related Neotropical taxa (e.g., birds [111], frogs [112]) and the numerous population size changes detected for all three species (Table 1) further underscore that the tropics are not necessarily demographically stable enclaves during climatic oscillations..

## Conclusions

Our study illustrates that glacial cycles have impacted both Nearctic and Neotropical milksnakes but that species did not share similar demographic responses by area. Most surprising, we found little evidence for expansions in the two most northerly distributed species, which were closest to the southern extent of the glacier during the LGM. In addition, results showed population declines for two of the tropical taxa. While it is likely that climatic events have contributed to the historical demographic patterns of these snakes, we cannot discount the possibility that unknown abiotic or biotic factors have altered populations. However, our examination of milksnake demography demonstrates that we cannot assume all temperate NW taxa have had similar responses to the last Ice Age. Furthermore, our study adds to the evidence that the tropics were not necessarily an area of stability during the Pleistocene and that populations underwent both expansions and declines. Based on these results we recommend using caution in generalizing demographic responses of taxa by region. Future studies that include greater numbers of loci, such as next-generation datasets comprised of hundreds of markers, combined with a comparative phylogeographic framework examining multiple taxonomic groups would be especially useful in achieving greater demographic resolution and determining whether species respond individually or if there are discernible patterns with respect to both phylogeny and biotic community.

## Supporting Information

S1 Table Individuals sequenced for demographics. Identification numbers correspond to those of Ruane et al. [62].
(DOCX)

S2 Table Total number of individuals sequenced (and used in EBSP analyses) for each locus and the number of individuals used only in DNAsp analyses. (DOCX)

S3 Table Approximate complete range size, sample range size, mean and median effective population size and number of size changes for six species of *Lampropeltis* based on the nuclear loci only. The 95% highest posterior density (HPD) is shown for each in parentheses; for number of size changes, species that had non-zero HPDs are indicated (*).
(DOCX)

S4 Table DNAsp results for each species and locus, including the number of haplotypes (h), haplotype diversity (Hd), number of segregating sites (S), nucleotide diversity (Pi), average number of nucleotide differences (k), Tajima's D, Fu and Li's D*, Fu and Li's F*, Ramos-Onsins and Rozas'S R2, and Harpending's raggedness index (rg). For all tests, the P-value (*P*) is included and for the R2 and rg results, the coalescent simulation results are shown, including the average R2 and rg, and the upper and lower confidence intervals for each (CI). Significant results are highlighted and in bold.
(DOCX)
