# Supplementary material for: Independent Demographic Responses to Climate Change among Temperate and Tropical Milksnakes (Colubridae: Genus Lampropeltis)
Source: PLoS One. 2015 Jun 17;10(6):e0128543. doi: 10.1371/journal.pone.0128543 (PMC4470684; doi:10.1371/journal.pone.0128543)
Supplement: S1 Table — Identification numbers correspond to those of Ruane et al. [62]. (DOCX) [file pone.0128543.s001.docx]

**S1 Table.** Individuals sequenced for demographics. Identification numbers correspond to those of Ruane et al. [62].

**ID # Genus Species State/Country County/State/Locality**

AMNH21940 *Lampropeltis abnorma* Mexico Guerrero

AMNH22617 *Lampropeltis abnorma* Mexico Veracruz

FTB1986 *Lampropeltis abnorma* Costa Rica Alajuela

FTB2317 *Lampropeltis abnorma* Nicaragua Central Nicaragua

FTB2320 *Lampropeltis abnorma* Nicaragua Central Nicaragua

FTB2321 *Lampropeltis abnorma* Honduras NW Honduras

FTB2322 *Lampropeltis abnorma* Honduras Southern Honduras

FTB2323 *Lampropeltis abnorma* Honduras Southern Honduras

FTB2386 *Lampropeltis abnorma* Honduras Choluteca

FTB2387 *Lampropeltis abnorma* Honduras Choluteca

FTB2411 *Lampropeltis abnorma* Costa Rica Guanacaste

FTB0797 *Lampropeltis elapsoides* FL Gulf

FTB1311 *Lampropeltis elapsoides* GA Lowndes

FTB1589 *Lampropeltis elapsoides* FL Palm Beach

FTB1591 *Lampropeltis elapsoides* FL Leon

FTB1593 *Lampropeltis elapsoides* FL Calhoun

FTB1597 *Lampropeltis elapsoides* FL Polk

FTB1598 *Lampropeltis elapsoides* FL Miami-Dade

FTB1606 *Lampropeltis elapsoides* FL Manatee

FTB1607 *Lampropeltis elapsoides* FL Manatee

FTB1615 *Lampropeltis elapsoides* FL Hendry

FTB1641 *Lampropeltis elapsoides* NC Brunswick

FTB1642 *Lampropeltis elapsoides* NC Jones

FTB1644 *Lampropeltis elapsoides* SC Jasper

FTB1645 *Lampropeltis elapsoides* GA Effingham

FTB1676 *Lampropeltis elapsoides* LA Tangipahoa

FTB1679 *Lampropeltis elapsoides* LA St. Tammany

FTB1691 *Lampropeltis elapsoides* MS Wilkinson

FTB1693 *Lampropeltis elapsoides* MS Stone

FTB1699 *Lampropeltis elapsoides* LA St. Tammany

FTB1701 *Lampropeltis elapsoides* FL Santa Rosa

FTB1702 *Lampropeltis elapsoides* LA West Feliciana

FTB1703 *Lampropeltis elapsoides* LA St. Tammany

FTB1755 *Lampropeltis elapsoides* SC Dorchester

FTB1756 *Lampropeltis elapsoides* AL Cleburne

FTB1794 *Lampropeltis elapsoides* AL Bibb

FTB1795 *Lampropeltis elapsoides* AL Bibb

FTB1974 *Lampropeltis elapsoides* SC Chesterfield

FTB1976 *Lampropeltis elapsoides* NC Scotland

FTB1977 *Lampropeltis elapsoides* SC Dorchester

FTB1978 *Lampropeltis elapsoides* NC Moore

FTB2109 *Lampropeltis elapsoides* NC Carteret

MHP9851 *Lampropeltis elapsoides* KY Lyon

DBS0562 *Lampropeltis gentilis* MT Carbon

DBS1406 *Lampropeltis gentilis* OK Atoka

DBS1452 *Lampropeltis gentilis* OK Pushmataha

DBS1535 *Lampropeltis gentilis* OK LeFlore

DBS2052 *Lampropeltis gentilis* AR Montgomery

FTB1531 *Lampropeltis gentilis* TX Val Verde

FTB1534 *Lampropeltis gentilis* TX Coleman

FTB1605 *Lampropeltis gentilis* LA Natchitoches

FTB1653 *Lampropeltis gentilis* NV Elko

FTB1690 *Lampropeltis gentilis* LA Iberville

FTB1700 *Lampropeltis gentilis* LA Jefferson

FTB1786 *Lampropeltis gentilis* UT Emery

FTB1789 *Lampropeltis gentilis* UT Uinta

FTB1797 *Lampropeltis gentilis* TX Grimes

FTB1798 *Lampropeltis gentilis* TX Burleson

FTB1805 *Lampropeltis gentilis* TX Brazos

FTB1806 *Lampropeltis gentilis* NM Torrance

FTB1817 *Lampropeltis gentilis* AZ Cochise

FTB1967 *Lampropeltis gentilis* AZ Coconino

FTB1971 *Lampropeltis gentilis* AZ Yavapai

FTB2224 *Lampropeltis gentilis* AZ Yavapai

LJV10749 *Lampropeltis gentilis* MT Carbon

LJV10753 *Lampropeltis gentilis* MT Carbon

LJV10782 *Lampropeltis gentilis* MT Yellowstone

MHP7910 *Lampropeltis gentilis* KS Rush

MHP8504 *Lampropeltis gentilis* KS Logan

MHP8549 *Lampropeltis gentilis* KS Russell

MHP8727 *Lampropeltis gentilis* SD Custer

SRSU6519 *Lampropeltis gentilis* TX Jeff Davis

TJC29  *Lampropeltis gentilis* OK Cherokee

FHGO2427 *Lampropeltis micropholis* Ecuador Pichincha

FHGO2663 *Lampropeltis micropholis* Ecuador Pichincha

FHGO2885 *Lampropeltis micropholis* Ecuador Pichincha

FHGO2906 *Lampropeltis micropholis* Ecuador Pichincha

FHGO2918 *Lampropeltis micropholis* Ecuador Pichincha

FHGO3021 *Lampropeltis micropholis* Ecuador Pichincha

FHGO3026 *Lampropeltis micropholis* Ecuador Pichincha

FHGO3090 *Lampropeltis micropholis* Ecuador Pichincha

FHGO4848 *Lampropeltis micropholis* Ecuador Pichincha

FHGO5766 *Lampropeltis micropholis* Ecuador Pichincha

FHGO5966 *Lampropeltis micropholis* Ecuador Pichincha

FHGO6217 *Lampropeltis micropholis* Ecuador Pichincha

FTB1985 *Lampropeltis micropholis* Costa Rica Cartago

QCAZR5576 *Lampropeltis micropholis* Ecuador Pichincha

QCAZR6321 *Lampropeltis micropholis* Ecuador Pichincha

USNM578513 *Lampropeltis micropholis* Panama Coclé

AMNH21975 *Lampropeltis polyzona* Mexico Guerrero

AMNH22074 *Lampropeltis polyzona* Mexico Guerrero

AMNH22361 *Lampropeltis polyzona* Mexico Oaxaca

AMNH22501 *Lampropeltis polyzona* Mexico Puebla

AMNH22785 *Lampropeltis polyzona* Mexico Oaxaca

AMNH22786 *Lampropeltis polyzona* Mexico Oaxaca

DGM2241 *Lampropeltis polyzona* Mexico Sonora

FMQ4000 *Lampropeltis polyzona* Mexico Hidalgo

FTB1568 *Lampropeltis polyzona* Mexico Colima

FTB1818 *Lampropeltis polyzona* Mexico Colima

FTB1821 *Lampropeltis polyzona* Mexico Puebla

FTB1964 *Lampropeltis polyzona* Mexico Sonora

FTB1965 *Lampropeltis polyzona* Mexico Sonora

HIR242 *Lampropeltis polyzona* Mexico Michoacán

JAC27958 *Lampropeltis polyzona* Mexico Colima

JAC29865 *Lampropeltis polyzona* Mexico Veracruz

JAC30084 *Lampropeltis polyzona* Mexico Colima

JAC30103 *Lampropeltis polyzona* Mexico Colima

JAC30303 *Lampropeltis polyzona* Mexico Colima

JAC30515 *Lampropeltis polyzona* Mexico Colima

JAC30516 *Lampropeltis polyzona* Mexico Colima

JAC30542 *Lampropeltis polyzona* Mexico Sinaloa

JAC30571 *Lampropeltis polyzona* Mexico Sonora

JAC30582 *Lampropeltis polyzona* Mexico Sinaloa

JAC30602 *Lampropeltis polyzona* Mexico Sinaloa

JAC30658 *Lampropeltis polyzona* Mexico Colima

JAC30659 *Lampropeltis polyzona* Mexico Colima

JLTV05 *Lampropeltis polyzona* Mexico Hidalgo

L177306 *Lampropeltis polyzona* Mexico Michoacán

PPC20  *Lampropeltis polyzona* Mexico Jalisco

PPC21  *Lampropeltis polyzona* Mexico Jalisco

FTB0442 *Lampropeltis triangulum* NY Suffolk

FTB0563 *Lampropeltis triangulum* VA Shenandoah

FTB0647 *Lampropeltis triangulum* WI Crawford

FTB0728 *Lampropeltis triangulum* WV Tucker

FTB0949 *Lampropeltis triangulum* NY Orange

FTB1512 *Lampropeltis triangulum* IL Jersey

FTB1521 *Lampropeltis triangulum* IL Washington

FTB1524 *Lampropeltis triangulum* IL Kankakee

FTB1525 *Lampropeltis triangulum* IL Iroquois

FTB1580 *Lampropeltis triangulum* MN Winona

FTB1581 *Lampropeltis triangulum* MN Winona

FTB1582 *Lampropeltis triangulum* IN Lake

FTB1584 *Lampropeltis triangulum* IN Lake

FTB1635 *Lampropeltis triangulum* MD Baltimore

FTB1638 *Lampropeltis triangulum* NJ Hunterdon

FTB1640 *Lampropeltis triangulum* MD Baltimore

FTB1678 *Lampropeltis triangulum* LA La Salle

FTB1680 *Lampropeltis triangulum* LA La Salle

FTB1687 *Lampropeltis triangulum* WI Walworth

FTB1697 *Lampropeltis triangulum* NY Tompkins

FTB1829 *Lampropeltis triangulum* MA Hampshire

FTB1839 *Lampropeltis triangulum* MO Jackson

FTB1840 *Lampropeltis triangulum* NY Sullivan

FTB1973 *Lampropeltis triangulum* PA Lackawanna

FTB1984 *Lampropeltis triangulum* WI Iowa

FTB2229 *Lampropeltis triangulum* MO Iron

FTB2230 *Lampropeltis triangulum* MO Iron

FTB2231 *Lampropeltis triangulum* MO Shannon

FTB2232 *Lampropeltis triangulum* MO Shannon

FTB2298 *Lampropeltis triangulum* OH Tusc

MHP9876 *Lampropeltis triangulum* TN Obion

MHP9886 *Lampropeltis triangulum* KY Hickman

MHP9887 *Lampropeltis triangulum* KY Menifee

YPM13969 *Lampropeltis triangulum* CT Fairfield
