# Supplementary material for: Independent Demographic Responses to Climate Change among Temperate and Tropical Milksnakes (Colubridae: Genus Lampropeltis)
Source: PLoS One. 2015 Jun 17;10(6):e0128543. doi: 10.1371/journal.pone.0128543 (PMC4470684; doi:10.1371/journal.pone.0128543)
Supplement: S2 Table — (DOCX) [file pone.0128543.s002.docx]

**S2 Table.** Total number of individuals sequenced (and used in EBSP analyses) for each locus and the number of individuals used only in DNAsp analyses.

**Species Locus # Individuals Sequenced # DNAsp Analyses**

*L. abnorma* 2CL3 11 9

*L. abnorma* 2CL4 11 10

*L. abnorma* 2CL8 11 8

*L. abnorma* CL4 11 11

*L. abnorma* CYTB 11 11

*L. abnorma* GAD2 11 11

*L. abnorma* LATCL 11 10

*L. abnorma* NT3 11 10

*L. abnorma* PRLR 10 9

*L. abnorma* SPTBN1 11 7

*L. abnorma* VIM56 11 10

*L. elapsoides* 2CL8 20 15

*L. elapsoides* CL4 31 31

*L. elapsoides* CYTB 32 32

*L. gentilis* 2CL8 19 6

*L. gentilis* CL4 29 24

*L. gentilis* CYTB 30 30

*L. micropholis* 2CL8 16 12

*L. micropholis* CYTB 12 11

*L. micropholis* GAD2 14 13

*L. micropholis* NT3 16 16

*L. micropholis* PRLR 12 12

*L. micropholis* SPTBN1 16 16

*L. polyzona* 2CL3 30 27

*L. polyzona* 2CL8 31 24

*L. polyzona* CL4 25 23

*L. polyzona* CYTB 31 23

*L. polyzona* GAD2 24 22

*L. polyzona* LATCL 23 22

*L. polyzona* NAV56 29 17

*L. polyzona* NT3 26 22

*L. polyzona* PRLR 30 22

*L. polyzona* SPTBN1 31 25

*L. polyzona* VIM56 30 23

*L. triangulum* 2CL8 20 20

*L. triangulum* CL4 33 29

*L. triangulum* CYTB 34 34
