# Supplementary material for: Independent Demographic Responses to Climate Change among Temperate and Tropical Milksnakes (Colubridae: Genus Lampropeltis)
Source: PLoS One. 2015 Jun 17;10(6):e0128543. doi: 10.1371/journal.pone.0128543 (PMC4470684; doi:10.1371/journal.pone.0128543)
Supplement: S3 Table — The 95% highest posterior density (HPD) is shown for each in parentheses; for number of size changes, species that had non-zero HPDs are indicated (*). (DOCX) [file pone.0128543.s003.docx]

**S3 Table.** Approximate complete range size, sample range size, mean and median effective population size and number of size changes for six species of *Lampropeltis* based on the nuclear loci only. The 95% highest posterior density (HPD) is shown for each in parentheses; for number of size changes, species that had non-zero HPDs are indicated (*)

**Species Population Size (Millions) Number of Population Size Changes**

**Mean, Median (95% HPD) Mean, Median (95% HPD)**

*L. triangulum*  3.3460, 1.6990 (9.543 x 10^4^ - 1.584 x 10^7^) 0.7, 0.0 (0-3)

*L. gentilis* 0.6780, 0.2800 (2.539 x 10^5^ - 1.715 x 10^7^) 1.5, 2.0 (0-4)

*L. elapsoides*  0.4070, 0.9200 (3.596 x 10^4^ - 9.325 x 10^6^) 0.7, 0.0 (0-2)

*L. polyzona* 0.0004, 0.0001 (2.082 x 10^-3^- 1.924 x 10^4^) 2.0, 2.0 (0-4)

*L. abnorma* 0.0034, 0.0047 (2.952 x 10^-4^- 4.352 x 10^2^) 0.7, 0.0 (0-2)

*L. micropholis* 0.1160, 0.6251 (5.674 x 10^2^ - 3.948 x 10^5^) 2.0, 2.0 (2-2)*
