# Supplementary material for: Independent Demographic Responses to Climate Change among Temperate and Tropical Milksnakes (Colubridae: Genus Lampropeltis)
Source: PLoS One. 2015 Jun 17;10(6):e0128543. doi: 10.1371/journal.pone.0128543 (PMC4470684; doi:10.1371/journal.pone.0128543)
Supplement: S4 Table — For all tests, the P-value (P) is included and for the R2 and rg results, the coalescent simulation results are shown, including the average R2 and rg, and the upper and lower confidence intervals for each (CI). Significant results are highlighted and in bold. (DOCX) [file pone.0128543.s004.docx]

**S4 Table.** DNAsp results for each species and locus, including the number of haplotypes (h), haplotype diversity (Hd), number of segregating sites (S), nucleotide diversity (Pi), average number of nucleotide differences (k), Tajima's D, Fu and Li's D*, Fu and Li's F*, Ramos-Onsins and Rozas'S R2, and Harpending's raggedness index (rg). For all tests, the P-value (*P*) is included and for the R2 and rg results, the coalescent simulation results are shown, including the average R2 and rg, and the upper and lower confidence intervals for each (CI). Significant results are highlighted and in bold.

**Species Locus h Hd S Pi k D *P* D* *P* F* *P* R2 *P* rg *P***

*L. abnorma* 2CL3 4 0.48 4 0.002 0.92 -0.62 P>0.10 1.12 P>0.10 0.74 P>0.10 0.12 0.13 0.35 0.72

*L. abnorma* 2CL4 4 0.61 2 0.002 0.67 -0.54 P>0.10 1.01 P>0.10 0.67 P>0.10 0.17 0.58 0.26 0.58

*L. abnorma* 2CL8 7 0.90 6 0.006 2.40 1.12 P>0.10 1.27 P>0.05 1.41 P>0.10 0.20 0.87 0.06 0.16

*L. abnorma* CL4 5 0.50 3 0.002 0.63 -0.60 P>0.10 -0.16 P>0.10 -0.32 P>0.10 0.11 0.16 0.11 0.13

*L. abnorma* CYTB 9 0.85 64 0.027 24.05 0.13 P>0.10 -0.35 P>0.10 -0.25 P>0.10 0.16 0.55 0.05 0.45

*L. abnorma* GAD2 7 0.84 5 0.003 1.50 0.28 P>0.10 1.18 P>0.10 1.07 P>0.10 0.15 0.65 0.10 0.27

*L. abnorma* LATCL 2 0.34 1 0.001 0.34 0.35 P>0.10 0.65 P>0.10 0.65 P>0.10 0.17 0.43 0.22 0.39

*L. abnorma* NT3 11 0.94 7 0.006 2.52 0.90 P>0.10 1.30 P>0.05 1.37 P>0.10 0.18 0.85 0.04 0.10

*L. abnorma* PRLR 2 0.21 1 0.001 0.21 -0.53 P>0.10 0.67 P>0.10 0.41 P>0.10 0.10 0.01* 0.38 0.72

*L. abnorma* SPTBN 6 0.85 8 0.004 2.91 0.59 P>0.10 1.37 P<0.05* 1.33 P>0.10 0.18 0.79 0.18 0.79

*L. abnorma* VIM56 3 0.36 3 0.001 0.72 -0.41 P>0.10 1.01 P>0.10 0.71 P>0.10 0.12 0.20 0.30 0.68

*L. elapsoides* 2CL8 3 0.58 6 0.012 3.09 2.97 P<0.01* 1.21 P>0.10 2.02 P<0.02* 0.26 1.00 0.49 0.99

*L. elapsoides* CL4 2 0.09 1 0.001 0.09 -0.71 P>0.10 0.53 P>0.10 0.19 P>0.10 0.05 0.01* 0.07 1.00

*L. elapsoides* CYTB 17 0.83 41 0.005 5.27 -1.76 P>0.05 -2.84 P<0.05* -2.93 P<0.05* 0.07 0.06 0.05 0.46

*L. gentilis* 2CL8 9 0.91 13 0.017 3.99 0.05 P>0.10 1.53 P<0.02* 1.26 P>0.10 0.15 0.80 0.11 0.72

*L. gentilis* CL4 11 0.87 10 0.009 3.02 0.98 P>0.10 1.40 P>0.05 1.49 P>0.05 0.15 0.18 0.03 0.06

*L. gentilis* CYTB 19 0.94 38 0.009 8.13 -0.61 P>0.10 -1.58 P>0.10 -1.49 P>0.10 0.10 0.28 0.03 0.28

*L. micropholis* 2CL8 5 0.50 6 0.003 1.11 -0.93 P>0.10 0.50 P>0.10 0.10 P>0.10 0.09 0.09 0.22 0.62

*L. micropholis* CYTB 7 0.66 43 0.008 7.87 -1.73 P>0.05 -1.73 P>0.10 -1.99 P>0.10 0.09 0.03* 0.10 0.77

*L. micropholis* GAD2 2 0.49 1 0.001 0.49 1.44 P>0.10 0.61 P>0.10 0.96 P>0.10 0.25 0.95 0.24 0.51

*L. micropholis* NT3 3 0.23 2 0.001 0.35 -0.61 P>0.10 0.80 P>0.10 0.46 P>0.10 0.09 0.13 0.43 0.63

*L. micropholis* PRLR 3 0.42 2 0.001 0.55 0.06 P>0.10 0.84 P>0.10 0.72 P>0.10 0.14 0.40 0.13 0.13

*L. micropholis* SPTBN 3 0.33 2 0.001 0.54 0.18 P>0.10 0.80 P>0.10 0.72 P>0.10 0.14 0.45 0.35 0.66

*L. polyzona* 2CL3 3 0.48 2 0.001 0.47 0.20 P>0.10 -0.91 P>0.10 -0.68 P>0.10 0.13 0.60 0.21 0.37

*L. polyzona* 2CL8 6 0.31 8 0.001 0.49 -1.84 P<0.05* -0.30 P>0.10 -0.93 P>0.10 0.04 0.04* 0.32 0.57

*L. polyzona* CL4 7 0.63 5 0.003 1.11 -0.07 P>0.10 1.10 P>0.10 0.87 P>0.10 0.11 0.51 0.03 0.01*

*L. polyzona* CYTB 18 0.97 129 0.049 39.57 0.35 P>0.10 0.58 P>0.10 0.60 P>0.10 0.15 0.77 0.04 0.97

*L. polyzona* GAD2 3 0.51 3 0.001 0.57 0.42 P>0.10 0.76 P>0.10 0.77 P>0.10 0.14 0.64 0.17 0.27

*L. polyzona* LATCL 3 0.28 3 0.001 0.29 -0.66 P>0.10 0.76 P>0.10 0.40 P>0.10 0.07 0.14 0.26 0.52

*L. polyzona* NAV56 8 0.85 9 0.005 1.87 - 0.45 P>0.10 1.36 P>0.05 0.94 P>0.10 0.10 0.35 0.05 0.11

*L. polyzona* NT3 19 0.95 16 0.015 5.51 1.31 P>0.10 1.61 P<0.02* 1.79 P<0.05* 0.17 0.97 0.02 0.02*

*L. polyzona* PRLR 8 0.44 8 0.002 0.75 -1.66 P>0.10 0.61 P>0.10 -0.13 P>0.10 0.05 0.03* 0.15 0.27

*L. polyzona* SPTBN 8 0.73 8 0.002 1.18 -1.10 P>0.10 -0.03 P>0.10 -0.43 P>0.10 0.08 0.16 0.06 0.12

*L. polyzona* VIM56 4 0.24 4 0.001 0.34 -1.45 P>0.10 1.01 P>0.10 0.31 P>0.10 0.04 >0.01* 0.37 0.59

*L. triangulum* 2CL8 8 0.75 10 0.017 2.47 0.15 P>0.10 1.40 P>0.05 1.18 P>0.10 0.12 0.64 0.07 0.36

*L. triangulum* CL4 7 0.42 7 0.004 1.23 -0.48 P>0.10 1.23 P>0.10 0.80 P>0.10 0.09 0.34 0.28 0.76

*L. triangulum* CYTB 18 0.94 31 0.007 6.51 -0.50 P>0.10 -0.72 P>0.10 -0.76 P>0.10 0.10 0.34 0.03 0.27
